# Avascular necrosis of the hip and diffuse idiopathic skeletal hyperostosis during long-term isotretinoin treatment of epidermolytic ichthyosis due to a novel deletion mutation in *KRT10*

**DOI:** 10.1111/bjd.13049

**Published:** 2014-08-05

**Authors:** RC Lamb, J Lang, A Terron-Kwiatowski, D Baty, WHI McLean, M Zamiri

**Affiliations:** 1 Department of Dermatology, University Hospital CrosshouseKilmarnock, U.K; 2 Department of Pathology, University Hospital CrosshouseKilmarnock, U.K; 3 East of Scotland Genetics Service, Ninewells HospitalDundee, U.K; 4 Centre for Dermatology and Genetic Medicine, Division of Molecular Medicine, University of DundeeDundee, U.K; 5 Alan Lyell Centre for DermatologyGlasgow, U.K

Dear Editor, Epidermolytic ichthyosis (EI; OMIM 113800), previously termed bullous congenital ichthyosiform erythroderma or epidermolytic hyperkeratosis, is a clinically heterogeneous disorder of keratinization. It is usually characterized by severe neonatal erythroderma, blistering and fragile skin, with the subsequent development of hyperkeratosis, predominantly in flexural areas. It is caused by mutations in either the *KRT1* or *KRT10* genes encoding the suprabasal keratins K1 and K10, respectively.^1^ Mutations are usually missense substitutions in the highly conserved alpha-helical rod domains of these keratins, which play a critical role in filament formation.^2^ We report a multigeneration kindred with EI due to a novel mutation in *KRT10*.

**Fig 1:**
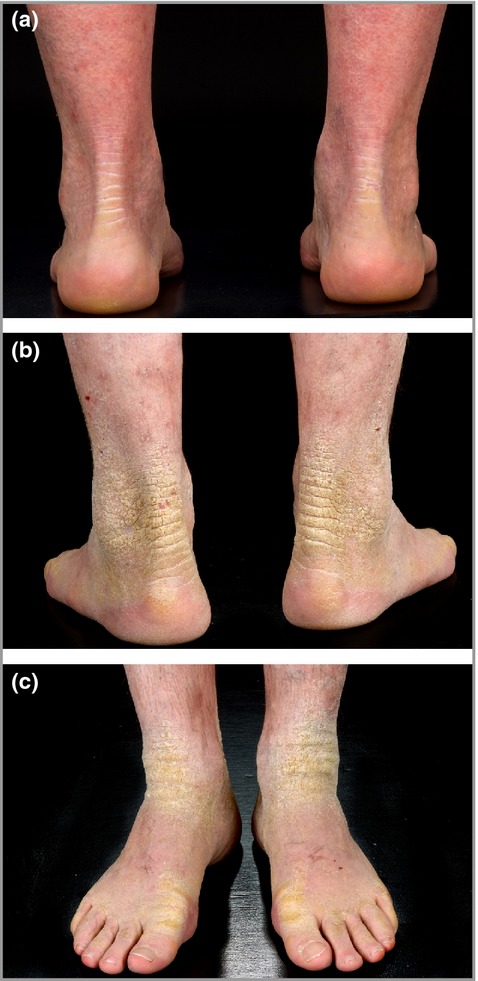
Family with epidermolytic ichthyosis. (a) Clinical picture of the proband: hyperkeratosis of nonplantar sites modified by oral retinoid therapy; (b, c) photographs of her father.

The proband was a 32-year-old woman from Shetland. She presented with widespread fine scale and erythema of her trunk and limbs, with a history of scaling and redness since birth but no blistering, erosions or collodion membrane reported. Clinical examination revealed widespread ichthyosis and erythema affecting the trunk and all four limbs, with more significant hyperkeratosis at the elbows, knees and ankles but relative sparing of palmoplantar skin (Fig. 1). Her father, uncle and grandmother were affected, and four further generations were reported to be affected. She had been maintained on oral isotretinoin 20–40 mg daily, thus modifying the clinical appearance, from the age of 13 years, but had recently developed lower back and hip pain. Radiographs of the lumbar spine and left hip demonstrated bridging osteophytes form T11 to L1, suggestive of diffuse idiopathic skeletal hyperostosis (DISH), and magnetic resonance imaging confirmed the presence of avascular necrosis of the left hip. This was successfully treated with core decompression of the left hip with improvement in the patient's pain.

A biopsy of affected skin of the upper limb was obtained from the proband and processed for light microscopy by standard methods. Structural analysis demonstrated acanthosis, marked overlying hyperkeratosis and vacuolar change of the upper epidermal cells with prominent clumping of keratohyaline granules.

**Fig 2:**
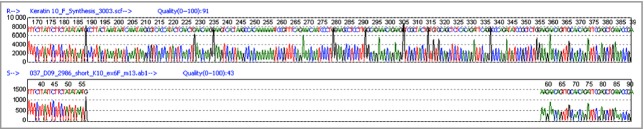
DNA sequencing of the *KRT10* gene in the proband. Sequencing reveals a novel heterozygous deletion extending from intron 5 into exon 6 (K10 c.1156–79_1243del), abolishing the exon 6 acceptor splice site resulting in a shorter aberrant keratin 10 (K10) protein lacking a sequence motif critically important for keratin filament assembly. The mutation was present in the proband's affected father but not in unaffected control samples.

Following informed consent, genomic DNA samples were obtained from blood samples from the proband and her father. Mutation analysis of the coding regions and splice sites of the *KRT1* and *KRT10* genes was performed by standard polymerase chain reaction (PCR) and Sanger sequencing methods using specific primers. Sequence analysis of *KRT10* revealed a previously unreported heterozygous deletion of 167 base pairs extending from intron 5 into exon 6 (c.1156–79_1243del), abolishing the intron 5 acceptor splice site (Fig. 2). This mutation was also present in the proband's affected father.

RNA was obtained from the proband's skin biopsy. Following reverse transcription by standard methods, reverse-transcriptase PCR was performed using primers flanking the deletion on exons 5 and 6 of *KRT10*. RNA analysis demonstrated that the *KRT10* c.1156–79_1243del deletion activates a cryptic splice site 96 base pairs downstream from the consensus intron 5–exon 6 splice site, resulting in an in-frame deletion of 32 amino acids, p.Lys386_Gln417, in the K10 protein. This truncated K10 protein, lacking the conserved helix termination motif, is likely to exert a dominant–negative effect on K1/K10 filament formation.

The keratin intermediate filament network is the main stress-bearing structure within the cytoplasm of epithelial cells. EI is caused by mutations in the keratin genes *KRT1* and *KRT10*, which confer structural integrity to suprabasal keratinocytes, with most reported mutations being heterozygous missense mutations.^3^ Approximately half of all cases of EI occur sporadically due to a spontaneous mutation.^2^ In inherited cases of EI, it is inherited mostly in a dominant mode, as in this pedigree, although recessive inheritance has been reported.^4^ Severe EI has been associated with mutations in the highly conserved helix boundary motifs, the helix initiation and termination peptides and the nonhelical H1 domain of K1 and K10.^3^ Mutations in the L1–2 linker of K1 or outside the helix boundary motifs, similar to those seen in mild epidermolysis bullosa simplex, have been described.^5^ A small number of insertion/deletion mutations and splice-site defects leading to larger in-frame deletions or rare dinucleotide alterations in *KRT10*, leading to substitution of two adjacent amino acids, have been described.^6^ Genotype–phenotype correlations in EI are very complex, and it is suggested that both the position of the mutation within K1 or K10 and the nature of the amino acid substitution specifically influence the phenotypic expression of the disease.^7^ Systemic retinoids should be used with caution in EI due to a risk of increased fragility and a tendency to blistering, although both topical and oral retinoids are thought to be more effective in those patients with EI with K10 mutations compared with K1. It is postulated that this may be due to the ability of patients with K10 mutations to tolerate better the downregulation of K2 caused by retinoids.^8^

To our knowledge, avascular necrosis of the femoral head has not previously been reported secondary to isotretinoin, and it is unclear whether this contributed to the findings in the proband. Avascular necrosis of the femoral head occurs due to interruption of the microcirculation of the femoral head resulting in ischaemia, and may occur spontaneously or in relation to treatment with glucocorticoids, hypertension, sickle cell disease, trauma or other causes. Avascular necrosis of the hip has been reported with retinoids (all-*trans*-retinoic acid) used to treat haematological conditions; however, affected cases have often received concomitant glucocorticoids.^9^ The long-term effects of oral retinoids remain under debate; however, experience of exposure to isotretinoin at this dose and for this length of time has been reported in the literature only rarely in comparison with other retinoids.^10^ Previously reported skeletal abnormalities with retinoid therapy include periosteal thickening, premature epiphyseal closure in children, osteoporosis, extraspinal tendon and ligament calcification, osteophytes and bony bridges between vertebrae as in the proband, in addition to DISH characterized by anterior spinal ligament calcification. However, there are few prospective studies on the skeletal effects of long-term systemic retinoids, and many of the bony changes reported are also prevalent in the general population. This finding adds to the evidence that *KRT10* mutations are the principal cause of autosomal dominant EI with palmoplantar sparing. It also highlights the possible side-effects of long-term oral retinoid treatment in these inherited conditions.
